# The Insight ToolKit image registration framework

**DOI:** 10.3389/fninf.2014.00044

**Published:** 2014-04-28

**Authors:** Brian B. Avants, Nicholas J. Tustison, Michael Stauffer, Gang Song, Baohua Wu, James C. Gee

**Affiliations:** ^1^Penn Image Computing and Science Laboratory, Department of Radiology, University of PennsylvaniaPhiladelphia, PA, USA; ^2^Department of Radiology and Medical Imaging, University of VirginiaCharlottesville, VA, USA

**Keywords:** registration, MRI, brain, open-source, death

## Abstract

Publicly available scientific resources help establish evaluation standards, provide a platform for teaching and improve reproducibility. Version 4 of the Insight ToolKit (ITK^4^) seeks to establish new standards in publicly available image registration methodology. ITK^4^ makes several advances in comparison to previous versions of ITK. ITK^4^ supports both multivariate images and objective functions; it also unifies high-dimensional (deformation field) and low-dimensional (affine) transformations with metrics that are reusable across transform types and with composite transforms that allow arbitrary series of geometric mappings to be chained together seamlessly. Metrics and optimizers take advantage of multi-core resources, when available. Furthermore, ITK^4^ reduces the parameter optimization burden via principled heuristics that automatically set scaling across disparate parameter types (rotations vs. translations). A related approach also constrains steps sizes for gradient-based optimizers. The result is that tuning for different metrics and/or image pairs is rarely necessary allowing the researcher to more easily focus on design/comparison of registration strategies. In total, the ITK^4^ contribution is intended as a structure to support reproducible research practices, will provide a more extensive foundation against which to evaluate new work in image registration and also enable application level programmers a broad suite of tools on which to build. Finally, we contextualize this work with a reference registration evaluation study with application to pediatric brain labeling.^1^

## 1. Introduction

As image registration methods mature—and their capabilities become more widely recognized—the number of applications increase (Rueckert et al., 1999; van Dalen et al., 2004; Miller et al., 2005; Shelton et al., 2005; Chen et al., 2008; Baloch and Davatzikos, 2009; Cheung and Krishnan, 2009; Peyrat et al., 2010; Fedorov et al., 2011; Kikinis and Pieper, 2011; Metz et al., 2011; Murphy et al., 2011). Consequently, image registration transitioned from being a field of active research, and few applied results, to a field where the main focus is translational. Image registration is now used to derive quantitative biomarkers from images (Jack et al., 2010), plays a major role in business models and clinical products (especially in radiation oncology) (Cheung and Krishnan, 2009), has led to numerous new findings in studies of brain and behavior (e.g., Bearden et al., 2007) and is a critical component in applications in pathology, microscopy, surgical planning, and more (Miller et al., 2005; Shelton et al., 2005; Floca and Dickhaus, 2007; Chen et al., 2008; Cheung and Krishnan, 2009; Peyrat et al., 2010; Kikinis and Pieper, 2011; Murphy et al., 2011). Despite the increasing relevance of image registration across application domains, there are relatively few reference algorithm implementations available to the community. Furthermore, these resources have become critical to setting performance standards in international challenges that evaluate “real world” registration scenarios (see, for instance, the SATA 2013 and BRATS 2013 challenges at MICCAI in Nagoya, Japan).

One source of benchmark methodology is the Insight ToolKit (ITK) (Yoo et al., 2002; Ackerman and Yoo, 2003), which marked a significant contribution to medical image processing when it first emerged at the turn of the millennium. Since that time, ITK has become a standard-bearer for image processing algorithms and, in particular, for image registration methods. In a review of ITK user interests, image registration was cited as the most important contribution of ITK (personal communication with Terry Yoo). Numerous papers use ITK algorithms as standard references for implementations of Demons registration and mutual information-based affine or B-Spline registration (van Dalen et al., 2004; Shelton et al., 2005; Floca and Dickhaus, 2007; Chen et al., 2008; Cheung and Krishnan, 2009). Multiple toolkits extend ITK registration methods in unique ways. Elastix provides very fast and accurate B-Spline registration (Klein et al., 2010; Murphy et al., 2011). The diffeomorphic demons is a fast/efficient approximation to a diffeomorphic mapping (Vercauteren et al., 2009). ANTs provides both flexibility and high average performance (Avants et al., 2011). The BRAINSFit algorithm is integrated into Slicer for user-guided registration (Kikinis and Pieper, 2011). Each of these toolkits has both strengths and weaknesses (Klein et al., 2010; Murphy et al., 2011) and was enabled by an ITK core.

The Insight ToolKit began a major refactoring effort in 2010. The refactoring aimed to both simplify and extend the techniques available in version 3.x with methods and ideas from a new set of prior work (Christensen et al., 1996; Rueckert et al., 1999; Jenkinson and Smith, 2001; Miller et al., 2005; Peyrat et al., 2010; Avants et al., 2011). To make this technology more accessible, ITK^4^ unifies the dense registration framework (displacement field, diffeomorphisms) with the low-dimensional (B-Spline, affine, rigid) framework by introducing composite transforms, deformation field transforms, and specializations that allowed these to be optimized efficiently. A sub-goal set for ITK^4^ was to simplify parameter setting by adding helper methods that use well-known principles of image registration to automatically scale transform components and set optimization parameters. ITK^4^ transforms are also newly applicable to objects such as vectors and tensors and will take into account covariant geometry if necessary. Finally, ITK^4^ reconfigures the registration framework to maximize multi-threading resources where possible. The revised registration framework within ITK is more thoroughly integrated across transform models, is thread-safe and provides broader functionality than in prior releases.

David Donoho once commented (in paraphrase) that academic publications are merely “advertisements” for the real work which is constituted by the “complete instruction set” that produces the results reported in the publication (Buckheit and Donoho, 1995). The first part of the remainder of this document will provide an “advertisement” for the ITK framework and summarize its evolution from ITK^3^ to ITK^4^. We then detail potential applications of this ITK^4^ framework in the context of a general nomenclature. While this work is indeed incomplete, in the sense of Donoho, we refer to source code and data when relevant. Furthermore, section 3.1 shows a series of reproducible examples of ITK^4^ in action. Several areas relevant to neuroinformatics are highlighted in these examples: optimal template construction, “challenging” registration scenarios involving brain mapping in the presence of lesions or resection, registration when initialization priors are weak, asymmetry analyses, functional MRI, and non-traditional registration strategies are all highlighted. We also establish performance benchmarks for the current ITK^4^ registration, in comparison to a method developed for ITK^3^, via a standard brain labeling task. Finally, we discuss future developments in the framework.

## 2. Materials and methods

### 2.1. Overview of the unified framework

The overall purpose of the registration refactoring for ITK^4^ was to simplify the user experience and to accelerate and improve performance. Here, we summarize how ITK^4^ works toward these goals.

#### 2.1.1. Core software components

**Figure 1:**
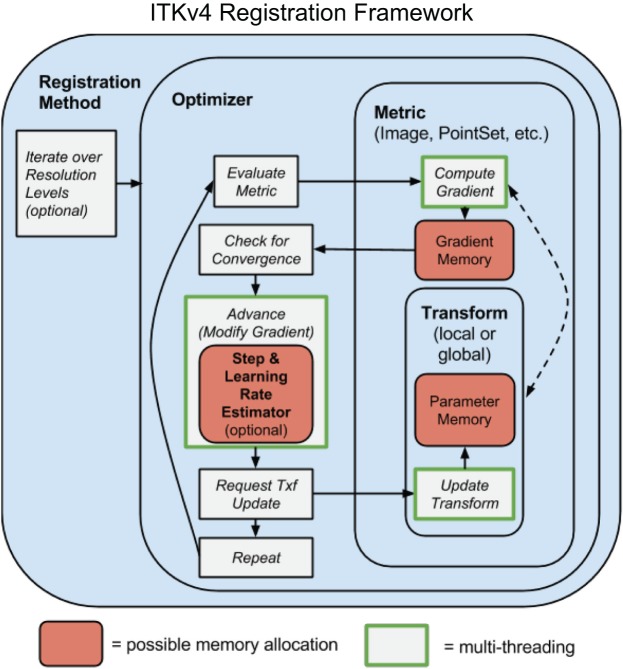
**A schematic overview of the prototypical ITK^4^ registration method**. This design is overall similar to that of ITK^3^. A few key components differ: (1) optimizers require that transforms update themselves; (2) metrics and optimizers are multi-threaded; (3) memory is shared across both optimizers and metrics, greatly increasing efficiency; (4) automated (usually hidden) parameter estimators are available; (5) transforms may include high-dimensional deformation fields. One additional difference (not shown) is that “fixed” images may also have a transformation, although this is not modified by the optimizer.

Figure 1 sketches the ITK^4^ architecture at a high level. Registration applications are known as “registration methods” as they were in ITK^3^. The methods, with source contained in ITK^4^'s RegistrationMethodsv4 directory, hold together the different subcomponents that make a working instantiation of a registration strategy. These subcomponents include the optimization technique (in the Optimizersv4 directory), the metric measuring the registration quality^2^ (the Metricsv4 directory), the images or other data objects that enter the metric and the parameters that are being optimized. The parameters are usually defined by a geometric transformation but may point to other relevant objects. Any of ITK^4^'s transformations may be optimized by the framework. New transformations, relative to ITK^3^, include the DisplacementField transforms that are useful for engendering Demons or B-Spline registration strategies. New VelocityField transforms are also available. A typical application developer would employ all of these components. A good starting point for new users who wish to see how these tools work together, in source code, is found in the tests. For instance, see the files itkTimeVaryingBSplineVelocityFieldImageRegistrationTest. cxx for an example of a B-Spline diffeomorphism application, itkSyNImageRegistrationTest.cxx to see SyN in ITK^4^ and itkSimpleImageRegistrationTest2.cxx for a more basic example.

Several usability goals spurred ITK^4^ development. We summarize these here.

#### 2.1.2. Image registration should be achievable in one step

**Figure 2:**
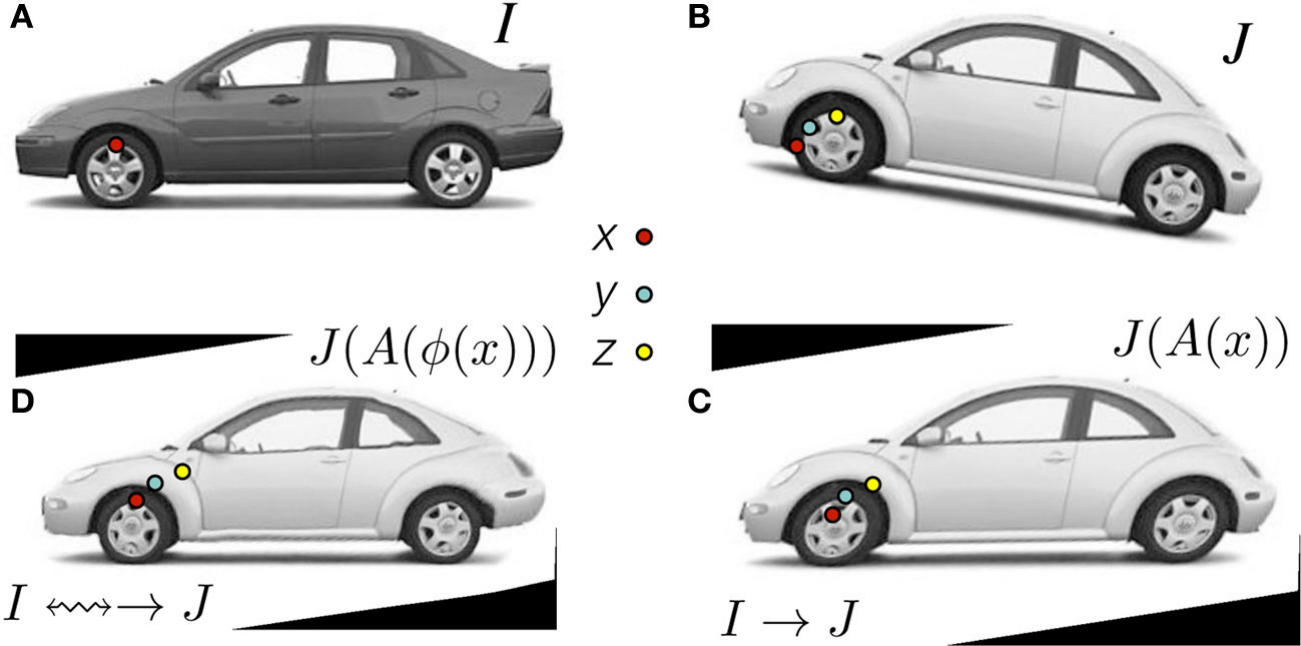
**Clockwise: Define *x* in Ω_*I*_ and *z* in Ω_*J*_ as the same material point but existing in different domains**. The point *y* is in a domain that is intermediate between Ω_*I*_ and Ω_*J*_. The standard approach in the ITKv4 registration framework is to map image *J* **(B)** to image *I* **(A)** by first identifying the linear transformation, →, between the images, shown in **(C)**. Second, we remove the shape (diffeomorphic) differences **(D)**. Consequently, we have a composite mapping, computed via the mutual information similarity metric, that identifies *I*(*x*) ≈_mi_ *J*(*A*(ϕ(*x*))) = *J*_Affine_(*y*) = *J*(*z*). The image *J*_Affine_(*y*) represents *J* after application of the affine transformation *A* i.e., *J*(*A*(*x*)). Code and data for this example are here.

This overarching goal is best illustrated by RegistrationMethodsv4 in which a user may string together a series of registration tools to perform (for instance) a translation registration, followed by an affine registration, followed by a diffeomorphic mapping each of which might use a different image similarity metric. The different transforms are accumulated in the new itkCompositeTransform which chains transforms together as in Figure 2. Thus, this framework provides unprecedented ability to perform complex and staged registration mappings. Furthermore, the frameworks automated parameter scaling, itkRegistrationParameterScalesEstimator, vastly reduces the difficulty of tuning parameters for different transform/metric combinations.

#### 2.1.3. ITK transforms should be unified

Each ITK^4^ transform now has either global support (affine transform) or local (or compact) support (a displacement field transform). If any map in a composite transform has global support then the composite transform has global support. Both “fixed” and “moving” images may have initial transforms. This allows one to reduce “registration bias” that may be induced by asymmetric interpolation (Yushkevich et al., 2010).

#### 2.1.4. Registration mappings should be applicable to a number of popular data types, including DTI

Our revisions to the ITK^3^ transform hierarchy validated and extended the ITK^3^ transforms for thread safety and applicability to not only vectors but also tensors. Reorientation steps necessary for diffusion tensor mappings are now included in ITK^4^.

#### 2.1.5. Affine and deformable similarity metrics should look as similar as possible

The Metricsv4 framework supports this goal in that it is as trivial to implement a mutual information Demons algorithm as it is to implement a sum of squared differences BSpline or affine registration algorithm. Thus, full plug-and-play support exists across transforms.

#### 2.1.6. Users should be able to combine multiple similarity metrics, some of which may operate on different data types

This is achievable with the existing itkMultiGradientOptimizerv4 through the multivariate itkObjectToObjectMultiMetricv4 or through the multi-channel traits (itkVectorImageToImageMetricTraitsv4) that allow metrics to deal with multi-channel pixels, all of which were contributed for ITK^4^. The itkObjectToObjectMultiMetricv4 was used in our winning entry of the SATA 2013 “dog leg” challenge.

#### 2.1.7. Optimizers and transformations should interact flexibly

Optimizersv4 includes optimizers that are applicable to both linear and deformable transformations, which include convergence monitoring and enable 2nd order optimization (itkQuasiNewtonOptimizerv4), multiple objective optimization (itkMultiGradientOptimizerv4), or global optimization (itkMultiStartOptimizerv4).

#### 2.1.8. GPU and multi-core acceleration will open up new applications for image registration

See GPUPDEDeformable for a GPU example. Furthermore, the new metric framework *N* cores to accelerate metric, gradient and optimization steps. A recent real-world application of the new Insight ToolKit implementation of the symmetric normalization algorithm showed a speed-up of almost a factor of six when comparing single core to eight core execution time. This speed-up is achieved by multi-threading the similarity metric, the gradient descent update, the regularization terms and the composition components of the method. Thus, every essential step exploits intrinsic parallelism in the algorithm. Decreased execution time means more rapid turnaround for users, faster turn-around in testing and higher throughput on large-scale computing tasks.

#### 2.1.9. Improve memory efficiency in optimization framework

Memory optimizations are critical for efficient use of large local transforms. In ITK^4^, transform parameters are no longer copied within the optimizer, but rather left in-place in transform. Metric gradient memory is shared between optimizer and metric, and modifications by the optimizer are done in place when possible.

Finally, we summarize ITK^4^ changes through quantitative metrics:

- Over 12 new multi-threaded image registration metrics are available in v4.
- Four application-level registration methods, with plug-and-play architecture, are available for high-level inclusion in projects such as Slicer and SimpleITK.
- All contributions are unit-tested and have greater than 85% code coverage, in accordance with ITK standards.
- A complete refactoring of the ITK transform hierarchy that makes transforms thread-safe, applicable to high-dimensional optimization and easily used in multi-core computing. Fourty-one classes, in total, were impacted by this refactoring.
- We added transparent vector support to two key interpolators that are used pervasively in ITK: the nearest neighbor and linear interpolators. We added two new Gaussian interpolators.
- An example of vector support for image metrics is in itkMeanSquaresImageToImageMetricv4VectorRegistrationTest.cxx.

Below we will discuss: (0) an organizing nomenclature matched to the ITK^4^ framework, (1) gradient-based optimization within the framework, (2) techniques to estimate optimization parameters for arbitrary metric and transformation combinations, (3) a ITK^4^ instance implementing generalized diffeomorphic matching, (4) several applications of the updated framework within different neuroinformatics-relevant domains.

### 2.2. Nomenclature

The nomenclature below designates an image registration algorithm symbolically. This nomenclature is intended to be a descriptive and technically consistent system for visually representing algorithms and applications of registration. Ideally, any standard algorithm can be written in the nomenclature below.

**Table:** 

*begin nomenclature definition*	
A physical point:	*x* ∈ Ω where Ω is the domain, usually of an image.
An image:	I : Ω^*d*^ → ℝ^*n*^ where *n* is the number of components per pixel and *d* is dimensionality. A second image is *J*.
Domain map:	ϕ : Ω_*I*_ → Ω_*J*_ where → may be replaced with any mapping symbol below.
Affine mapping:	↔ a low-dimensional invertible transformation: affine, rigid, translation, etc.
Affine mapping:	→ designates the direction an affine mapping is applied.
Deformation field:	⇝ deformation field mapping *J* to *I*. May not be invertible.
Spline-based mapping:	$\underset{b}{⇝}$ e.g., B-Spline field mapping *J* to *I*.
Diffeomorphism:	Represented as ↭, these are differentiable maps with differentiable inverse. Ideally, the algorithm should output the inverse and forward mapping.
Composite mapping:	ϕ = ϕ_1_(ϕ_2_(*x*)) is defined by ↭ → where ϕ_2_ is of type ↭.
Not invertible:	↮ indicates a mapping that is not invertible.
Perform image warping:	As an example, → *J* represents the application of an affine transform → to image *J* such that → *J* = *J*(*A*(*x*)).
Similarity measure:	$\underset{s}{\approx }$ or ≈_*s*_ indicates the metric *s* that compares a pair of images.
*end nomenclature definition*	

We would then write a standard Demons registration application that maps one image, *J*, into the space of *I* (presumably a template) as:

I⇝→J     which symbolizes     I≈J(A(ϕ(x))),

with *A* an affine mapping and ϕ a generic deformation. The notation means that the algorithm first optimizes an affine mapping, →, between *J* and *I*. This is followed by a deformation in the second stage, ⇝, from → *J* to *I*. In terms of transformation composition, we would write ⇝ → *J* = *J*_*w*_(*x*) = *J*(ϕ_Affine_ (ϕ_Demons_(*x*))) where *J*_*w*_ is the result of warping *J* to *I*. The ϕ are the specific functions corresponding to the schematic arrows. Note, also, that the tail of the arrow indicates the transform's domain. The arrowhead indicates its range. Finally, we denote the similarity metric as ≈ which indicates a sum of squared differences (the default similarity metric). ITK^4^ supports metrics such as mutual information, $\underset{\text{mi}}{\approx }$, or cross-correlation, $\underset{\text{cc}}{\approx }$. We will use this nomenclature to write schematics for registration applications in the following sections. ^3^

### 2.3. Optimization framework

The general ITK^4^ optimization criterion is summarized as:

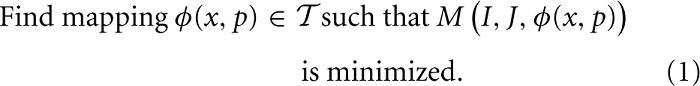



While, for functional mappings, this formulation is not strictly correct, the practical implementation of even high-dimensional continuous transformations involves parameterization. The space 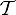 restricts the possible transformations over which to optimize the mapping ϕ. The arguments to ϕ are its parameters, *p*, and the spatial position, *x*. Note that, in ITK^4^, the image *I* may also contain a mapping, although it is not directly optimized in most cases. As will be seen later in the document, this mapping may also be used within large deformation metrics.

The similarity metric, *M*, is perhaps the most critical component in image registration. Denote a parameter set as *p* = (*p*_1_, *p*_2_ … *p*_*n*_). The metric (or comparison function between images) is then defined by *M*(*I, J*, ϕ(*x, p*)). For instance, *M* = ‖*I(x)* − *J*(ϕ(*x, p*))‖^2^ i.e., the sum of squared differences (SSD) metric. Its gradient with respect to parameter *p*_*i*_ is (using the chain rule),

(2) $$M_{p_{i}}=\frac{\partial M}{\partial p_{i}}=\frac{\partial M}{\partial J}\frac{\partial J\left(\phi (x,p)\right)}{\partial \phi }{\frac{\partial \phi }{\partial p_{i}}}^{T}{|}_{x}.$$

This equation provides the metric gradient specified for sum of squared differences (at point *x*) but similar forms arise for the correlation and mutual information (Hermosillo et al., 2002). Both are implemented in ITK^4^ for transformations with local and global support. The $\frac{\partial J(\phi (x,p))}{\partial \phi }$ term is the gradient of *J* at ϕ(*x*) and $\frac{\partial \phi }{\partial p_{i}}$ is the Jacobian of the transformation taken with respect to its parameter. The transform ϕ(*x, p*) may be an affine map i.e., ϕ(*x, p*) = *Ax* + *t* where *A* is a matrix and *t* a translation. Alternatively, it may be a displacement field where ϕ(*x, p*) = *x* + *u*(*x*) and *u* is a vector field. In ITK^4^, both types of maps are interchangeable and may be used in a composite transform to compute registrations that map to a template via a schematic such as $I\approx \to J,I\underset{\text{mi}}{\approx }\underset{b}{⇝}\to J,I\underset{\text{cc}}{\approx }↭\to J$ or, mixing similarity metrics, *I* ≈_cc_ ↭ ≈_mi_ → *J*_*i*_.

The most commonly used optimization algorithm for image registration is gradient descent, or some variant. In the above framework, the gradient descent takes on the form of

$$\phi \left(p_{\text{new}},x\right)=\phi \left(p_{\text{old}}+\lambda \;\left[\frac{\partial M}{\partial p_{1}},\cdots ,\frac{\partial M}{\partial p_{n}}\right],x\right),$$

where λ is the overall learning rate and the brackets hold the vector of parameter updates.

In addition to basic gradient descent, we implement non-linear gradient descent optimization strategies which combine the conjugate gradient or gradient descent method with line search. In ITK^4^, we implement the classic *golden section* approach to identifying the optimal gradient step-size at each iteration. The generic conjugate gradient approach is performed via:

(3) $$\begin{array}{l} \;\gamma =\frac{\|\nabla M_{t}-\|\nabla M_{t\;-\;1}{\|}^{2}{\|}^{2}}{\|\nabla M_{t\;-\;1}{\|}^{2}},\\ CG_{t}=\nabla M_{t}+\gamma CG_{t\;-\;1}, \end{array}$$

where *CG* is the conjugate gradient. The golden section line search determines the specific weight, ϵ_opt_, of the update to the current parameters such that

$$p_{\text{new}}=p_{\text{old}}+CG_{t}\epsilon_{\text{opt}}.$$

Note that a naive application of gradient descent will not produce a smooth change of parameters for transformations with mixed parameter types. For instance, a change, Δ, to parameter *p*_*i*_ will produce a different magnitude of impact on ϕ if *p*_*i*_ is a translation rather than a rotation. Thus, we develop an estimation framework that sets “parameter scales” (in ITK parlance) which, essentially, customize the learning rate for each parameter. The update to ϕ via its gradient may also include other steps (such as Gaussian smoothing) that project the updated transform back to space 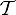. Multi-threading is achieved in the gradient computation, transformation update step and (if used) the regularization by dividing the parameter set into computational units that correspond to contiguous sub-regions of the image domain.

In terms of code, the Jacobian, $\frac{d\phi }{dp}{|}_{x}$, is calculated at a physical point using the function *ComputeJacobianWithRespectToParameters(mappedFixedPoint, Jacobian)*. Note that it is evaluated at point *x* not at point ϕ(*x, p*). We then use the function *ComputeMovingImageGradientAtPoint(mappedMovingPoint, mappedMovingImageGradient)* to compute the moving image gradient when there is no pre-warping. *ComputeMovingImageGradientAtPoint* uses central differences (or a gradient filter) in the moving image space to compute the image gradient, $\frac{dJ(\phi (x,p))}{d\phi }$.

If one is doing pre-warping, then we have an index access to the warped moving image. We compute the warped image *J* as *J*_*w*_(*x*) = *J*(ϕ(*x, p*)). Then,

(4) $$\begin{array}{l} \;\frac{dJ_{w}}{dx}=\frac{dJ\left(\phi \left(x,p\right)\right)}{d\phi }\frac{d\left(\phi (x,p)\right)}{dx}\\ \frac{dJ\left(\phi (x,p)\right)}{d\phi }=\frac{dJ_{w}}{dx}{\frac{d\left(\phi (x,p)\right)}{dx}}^{-1}\text{​}. \end{array}$$

In code, we use *ComputeMovingImageGradientAtIndex(index, mappedMovingImageGradient)* to get $\frac{dJ_{w}}{dx}$ and transform this image gradient via the inverse Jacobian by calling *mappedMovingImageGradient= TransformCovariantVector(mappedMovingImageGradient, mappedMovingPoint)*.

### 2.4. Diffeomorphic mapping with arbitrary metrics

The framework proposed above, in general form, encompasses both classic affine mapping as well as more recent large deformation strategies. Beg proposed the Large Deformation Diffeomorphic Metric Mapping (LDDMM) algorithm (Miller et al., 2005) which minimizes the sum of squared differences criterion between two images. LDDMM parameterizes a diffeomorphism through a time varying velocity field that is integrated through an ODE. In ITK^4^, we implement an alternative to LDDMM that also uses a time varying field and an ODE but minimizes a more general objective function:

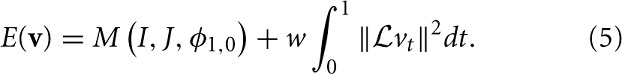



**Figure 3:**
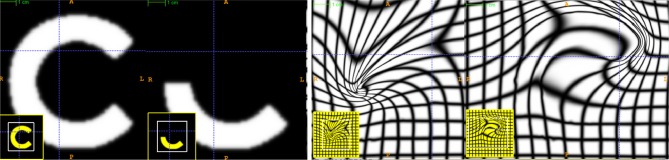
**An ITK diffeomorphic mapping of the type *I* ↭ *J***. The “C” and 1/2 “C” example illustrate the large deformations that may be achieved with time varying velocity fields. In this case, the moving (deforming) image is the 1/2 “C.” The right panels illustrate the deformed grid for the transformation of the “C” to 1/2 “C” (middle right) and its inverse mapping (far right) which takes the 1/2 “C” to the reference space. The unit time interval is discretized into 15 segments in order to compute this mapping. 15^*^5 integration steps were used in the Runge-Kutta ODE integration over the velocity field. A two core MacBook Air computed this registration in 110 s. The images each were of size 150 × 150. See C for a reproducible example of this registration and the data. In addition, we provide an example of how the Jacobian determinant is computed from the deformation field resulting from this registration via an *ANTs* program CreateJacobianDeterminantImage.

This is an instance of Equation (1) where *w* is a scalar weight and ϕ_1, 0_ is a standard integration of the time-varying velocity field, *v*_*t*_, which is regularized by the linear operator 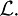. ITK^4^ uses Gaussian smoothing which is the Green's kernel for generalized Tikhonov regularization (Nielsen et al., 1997). This objective is readily optimized using an approach that is similar to that proposed by Beg. Generalization of the LDDMM gradient for other metrics basically follows (Hermosillo et al., 2002) with a few adjustments to accomodate diffeomorphic mapping. Figure 3 shows an ITK result on a standard example for large deformation registration. We will evaluate this diffeomorphic mapping, along with parameter estimation, in a later section.

### 2.5. Parameter scale estimation

We choose to estimate parameter scales by analyzing the result of a small parameter update on the change in the magnitude of physical space deformation induced by the transformation. The impact from a unit change of parameter *p*_*i*_ may be defined in multiple ways, such as the maximum shift of voxels or the average norm of transform Jacobians (Jenkinson and Smith, 2001). Denote the unscaled gradient descent update to *p* as Δ*p*. The goal is to rescale Δ*p* to *q* = *s* · Δ*p*, where *s* is a diagonal matrix diag(*s*_1_, *s*_2_ … *s*_*n*_), such that a unit change of *q*_*i*_ will have the same impact on deformation for each parameter *i* = 1 … *n*.

As an example, we want ‖ϕ(*x, p*_new_) − ϕ(*x, p*_old_)‖ = *constant* regardless of which of the *i* parameters is updated by the unit change. The unit is an epsilon value e.g., 1.e-3. Rewrite $\left[\frac{\partial M}{\partial p_{1}},\cdots ,\frac{\partial M}{\partial p_{n}}\right]$ as $\frac{\partial M}{\partial J}\frac{\partial J(\phi (x,p))}{\partial \phi }\left[\frac{\partial \phi }{\partial p_{1}},\cdots ,\frac{\partial \phi }{\partial p_{n}}\right]$. To determine the relative scale effects of each parameter, *p*_*i*_, we can factor out the constant terms on the outside of the bracket. Then the modified gradient descent step becomes $\text{diag}(s)\frac{\partial \phi }{\partial p}$. We identify the values of diag(*s*) by explicitly computing the values of ‖ϕ(*x, p*_new_) − ϕ(*x, p*_old_)‖ with respect to an ϵ change. A critical variable, practically, is which *x* to choose for evaluation of ‖ϕ(*x, p*_new_) − ϕ(*x, p*_old_)‖. The corners of the image domain work well for affine transformations. In contrast, local regions of small radius (approximately 5) work well for transformations with local support. Additional work is needed to verify optimal parameters for this new ITK^4^ feature. However, a preliminary evaluation is performed in the results section. The new parameter scale estimation effectively reduces the number of parameters that the user must tune from *k* + 1 (λ plus the scales for each parameter type where there are *k* types) to only 1, the learning rate.

The learning rate, itself, may not be intuitive for a user to set. The difficulty—across problem sets—is that a good learning rate for one problem may result in a different amount of change per iteration in another problem. Furthermore, the discrete image gradient may become invalid beyond one voxel. Thus, it is good practice to limit a deformation step to one voxel spacing (Jenkinson and Smith, 2001). We therefore provide the users the ability to specify the learning rate in terms of the *maximum physical space change per iteration*. As with the parameter scale estimation, the domain over which this maximum change is estimated impacts the outcome and similar practices are recommended for both cases. This feature is especially useful for allowing one to tune gradient descent parameters without being concerned about which similarity metric is being used. That is, it effectively rescales the term λ∂*M*/∂*p* to have a consistent effect, for a given λ, regardless of the metric choice. In combination with our non-linear conjugate gradient approach (our current optimization of choice for linear registration), this strategy drastically reduces the parameter setting burden for users.

## 3. Results

### 3.1. Example applications of the ITK^4^ framework

As part of our work in ITK refactoring, we built, in parallel to library programming, an application interface that allows high-level access to the deep layers of ITK registration. These currently exist in the *Advanced Normalization Tools* (ANTs) software (link). While ANTs still serves as intermediate (vs. direct) access-point to these tools, it provides a high-degree of customization possibilities simply through a command line interface and scripting. Therefore, a user is not required to write new low-level (interestingly, C++ is now considered “low-level”) software.

Despite its relative youth, the ANTs wrapping of ITK functionality has been employed with notable success in recent public, unbiased, international evaluation studies. ANTs was instrumental to a first-place finish in SATA 2013 in two of three categories (based on the median performance) where the ANTs approach was considerably simpler than that employed by close finishers. While the evaluation of deep-gray matter registration showed relatively subtle differences, the ANTs solution to the multivariate canine leg data outclassed all other entrants. Notably, the ANTs solution used a multiple metric approach that simultaneously compared two modalities during registration as in Avants et al. (2008). In the cardiac data, the ANTs solution was the only one that was fully automated resulting in a ≈15% performance loss which can easily be overcome by a modicum of user intervention. Furthermore, ANTs/ITK-based methods finished a clear first-place in the BRATS 2013 challenge. Our entry used intensity asymmetry as a key feature to segment brain tumors based on multiple modality MRI. Thus, these methods are within the leading ranks of image registration methodologies as evaluated in recent work as well as in the more traditional brain (Klein et al., 2010) and lung CT (Murphy et al., 2011) studies.

**Figure 4:**
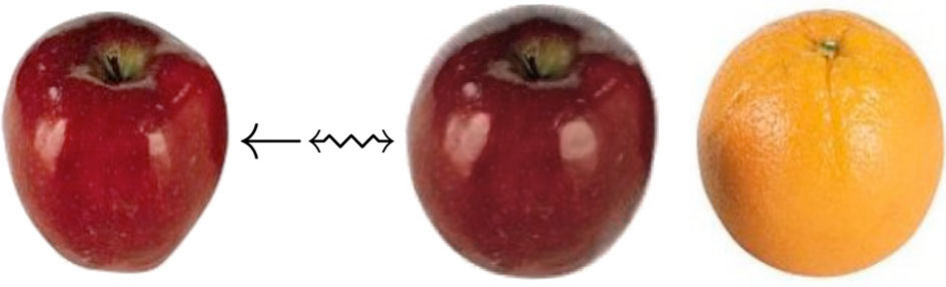
**This RGB image registration example employs ITK^4^ code that repurposes a scalar metric (itkMeanSquaresImageToImageMetricv4) for multichannel registration**.

The ANTs contribution is valuable, in part, because of the tremendous range of registration problems that exist in neuroinformatics and biomedical imaging in general. While it is not possible to solve all registration problems with a general framework, one cannot afford to invent new solutions for every instance one encounters. Our general optimization-driven strategies have proven to be invaluable to setting performance standards in a variety of application domains. In this section, we highlight some of the lesser known capabilities of ANTs and ITK^4^ with reproducible examples that include data and specific commands to ANTs and/or ITK. A list of these examples follows:

1. The Basic Brain Mapping example shows how one can map two “whole head” T1-weighted MRIs where one is a template that contains a researcher's prior knowledge defining the “interesting” parts of the image. Within ITK^4^, this domain knowledge is used to focus the Metricsv4 on only those parts of the image while masking the remainder. Furthermore, a second part of this example shows how the ITK composite transform may be used to initialize new registration solutions as well as how masking functionality may be employed to ignore information that is irrelevant or obstructive to the registration optimization. We have previously employed these strategies in brain mapping with lesions (Avants et al., 2008; Kim et al., 2008, 2010, 2013; Tustison et al., 2011).
2. We use updated ITK methods in template construction with a reproducible example based on face and brain data: ANTs template construction. This work has been employed in different species, age-ranges, and imaging modalities. The resulting template is an image that captures the expected shape and appearance as defined by the population sample, transformation model and intensity comparison metric.
3. A large deformation example implementing the classic “letter C” example provided, originally, by Gary Christensen. While extremely flexible, these algorithms have not found a unique identity in terms of translational applications yet remain of theoretical interest. This example shows a user how to define the parameters of a registration based on optimizing a time varying velocity field.
4. We present a separate example of how to compute landmark-based registration error. ITK uses LPS coordinates to represent physical space. If you need to convert landmarks to physical space, see the discussion here: LPS physical space. We have an example illustrating how to change point coordinates and apply ITK transforms to landmarks here. This exercise can be useful for landmark-based registration or in evaluating registration accuracy.
5. We show how to perform motion correction to time series data here although we do not claim this approach is optimal. The method registers each frame from a 4D time series to a fixed reference image and stores the resampled set in a new 4D image. All transformation parameters are stored in a corresponding csv file.
6. An advanced example with heavy use of statistics via *R* and *ANTsR* is in a study of public test-retest fmri data. This study is not published and may be subject to change.
7. The classic car example shown in ANTs talks is here. This illustrates the benefit of mutual information in deformably mapping wild-type images and highlights the fact that ITK^4^ applications exist outside of medical imaging.
8. A basic multistart example. A more advanced example for brain mapping with a template mask is available in *antsCorticalThickness.sh*. These optimization methods overcome local minima by running registration searches from a variety of starting points and greedily storing the best solution.
9. This asymmetry analysis example uses the “virtual domain” feature to reduce bias caused by mapping an image asymmetrically to a reference. Note that we measure the point-wise asymmetry, in this example, via the Jacobian determinant image as in Kim et al. (2008). If one repeats this analysis across a population—and maps the Jacobian measurements of asymmetry to a common space—then one may perform a statistical analysis of population-level asymmetry. Longitudinal analysis and asymmetry analysis potentially suffer from the same confound Yushkevich et al. (2010). A related longitudinal mapping script is here.
10. We also show how manual labelings can be used to restrict registration in a challenging registration scenario (this example will be improved in the future): registration guided by (crude) labels.
11. A simple orange to apple RGB image registration example for color images is listed at: itkMeanSquaresImageToImageMetricv4VectorRegistrationTest. If one compiles the ITK tests, then this example can be run to produce Figure 4.

These examples cover many applications for which no “ground truth” evaluation data exists. The next section seeks to add some quantitative reference to these examples. First, we show flexibility and consistency of our framework in a simple example comparing registration with a variety of metrics and a consistent parameter set. Second, we quantify the benefit of ITK^4^ registration in comparison to a method implemented based upon ITK^3^ registration technology.

### 3.2. Evaluation

We first investigate the ability of our automated parameter estimation to facilitate parameter tuning across metrics. Second, we compare ITK^4^ and ITK^3^ registration implementations with respect to a standard automated brain labeling task.

#### 3.2.1. Parameter estimation across metrics

**Figure 5:**
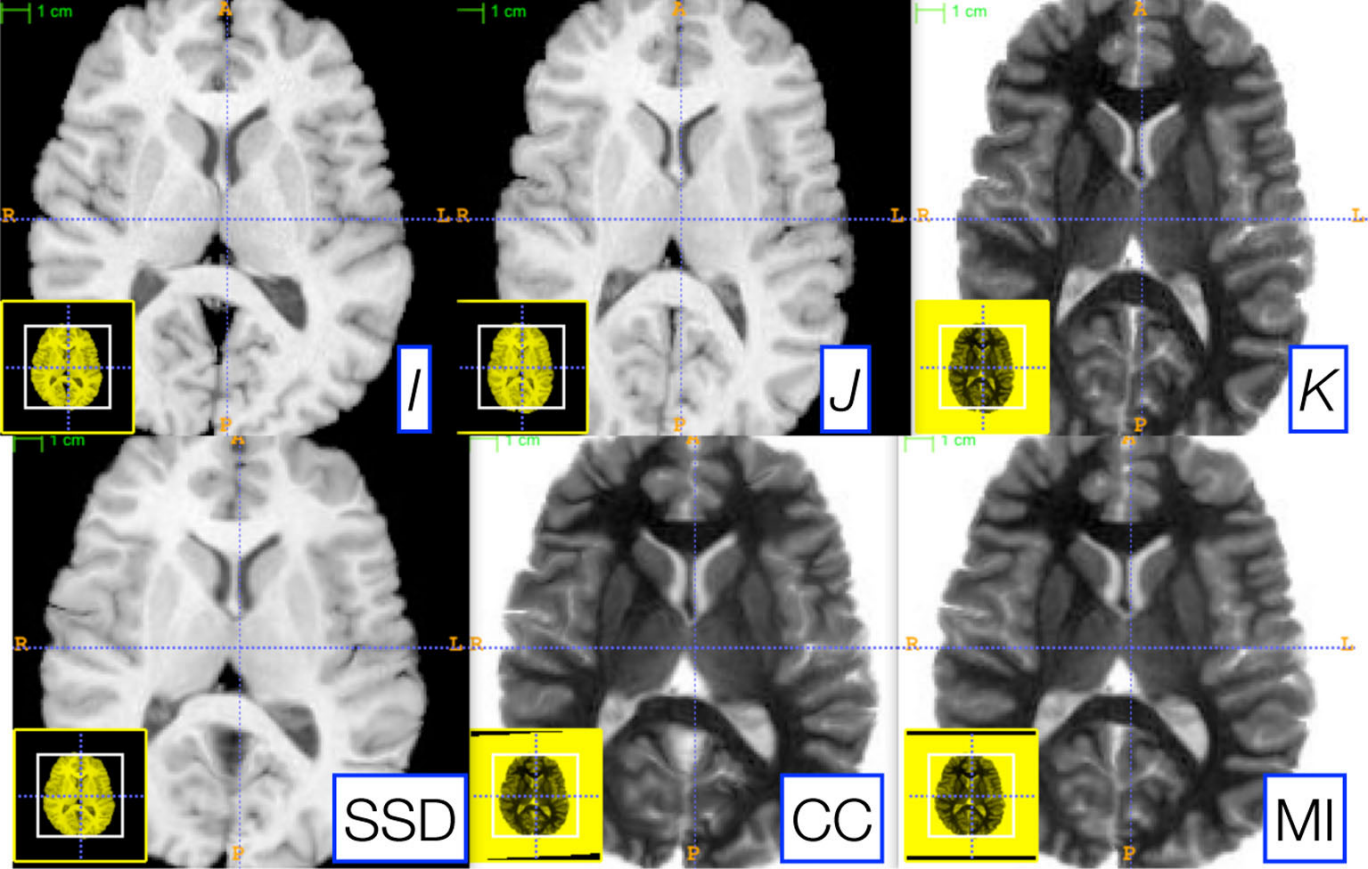
**Three reference images, *I* (left), *J* (middle top), and *K* (right top), are used to illustrate the robustness of our parameter scale estimation for setting consistent parameters across both metrics and transform types**. *K* is the negation of *J* and is used to test the correlation and mutual information registrations. We optimized, by hand, the step-length parameters for one metric (the sum of squared differences) for both the affine and deformable case. Thus, two parameters had to be optimized. We then applied these same parameters to register *I* and *K* via both correlation and mutual information. The resulting registrations (bottom row) were all of similar quality. Further, the same metric is used for both affine and diffeomorphic mapping by exploiting the general optimization process given in Equation (1).

ITK^4^ provides similarity metrics that may be applied for both deformable and affine registration. In a previous section, we provided a parameter estimation strategy that is applicable to both deformable and affine transformations with arbitrary metrics. Denote images *I, J, K*, where the latter two are “moving” images, and *K* is an intensity-inverted version of *J*. We then evaluate the following schema,

$$I\approx ↭\to J,\;\;\;\;\;\;\;I\approx_{\text{cc}}↭\to K,\;\;\;\;\;\;\;I\approx_{\text{mi}}↭\to K$$

where, for each schematic, we use the corresponding metric for both affine and diffeomorphic mapping. Furthermore, we keep the same parameters for each registration by exploiting parameter scale estimators. Figure 5 shows the candidate images for this test.

As shown in Figure 5, very similar results are achieved for each schematic without additional parameter tuning. To determine this quantitatively, we perform registration for each schematic and then compare the Dice overlap of a ground-truth three-tissue segmentation. For each result, we have the Dice overlap of dark tissue (cerebrospinal fluid, CSF), medium intensity tissue (gray matter) and bright tissue (white matter). For the mean squares metric, we have: 0.588, 0.816, and 0.90; for CC, we have: 0.624, 0.786, 0.882; for MI, we have: 0.645, 0.779, 0.858. Mutual information does best for the CSF while mean squares does best for other tissues. CC performs in the mid-range for all classes of tissue. Thus, a single set of tuned parameters provides a reasonable result for an affine plus diffeomorphic mapping across three different metrics. While improvement might be gained by further tuning for each metric, this result shows that our parameter estimation method achieves the goal of reducing user burden.

#### 3.2.2. Automated brain labeling task

All *R* and bash analysis scripts for this section are here: https://github.com/stnava/ITKv4Documentation/tree/frontiers/scripts. The ITK^4^ core functionality formed the heart of the reference results provided for the SATA2013 challenge at MICCAI 2013. In this sense, these methods have been heavily evaluated on both basic brain mapping challenges (SATA2013's diencephalon challenge in which ITK^4^-based methods finished first), multivariate registration challenges (the canine MRI / dog leg challenge of SATA2013 in which ITK^4^-based methods were overwhelmingly the top finisher) and in the cardiac challenge (in which ITK^4^-based methods were the only fully automated approach). However, for completeness, we provide an additional evaluation here which focuses on comparison to a ITK^3^ method, BRAINSFit, in a different dataset than previously used to evaluate ANTs or ITK^4^.

**Figure 6:**
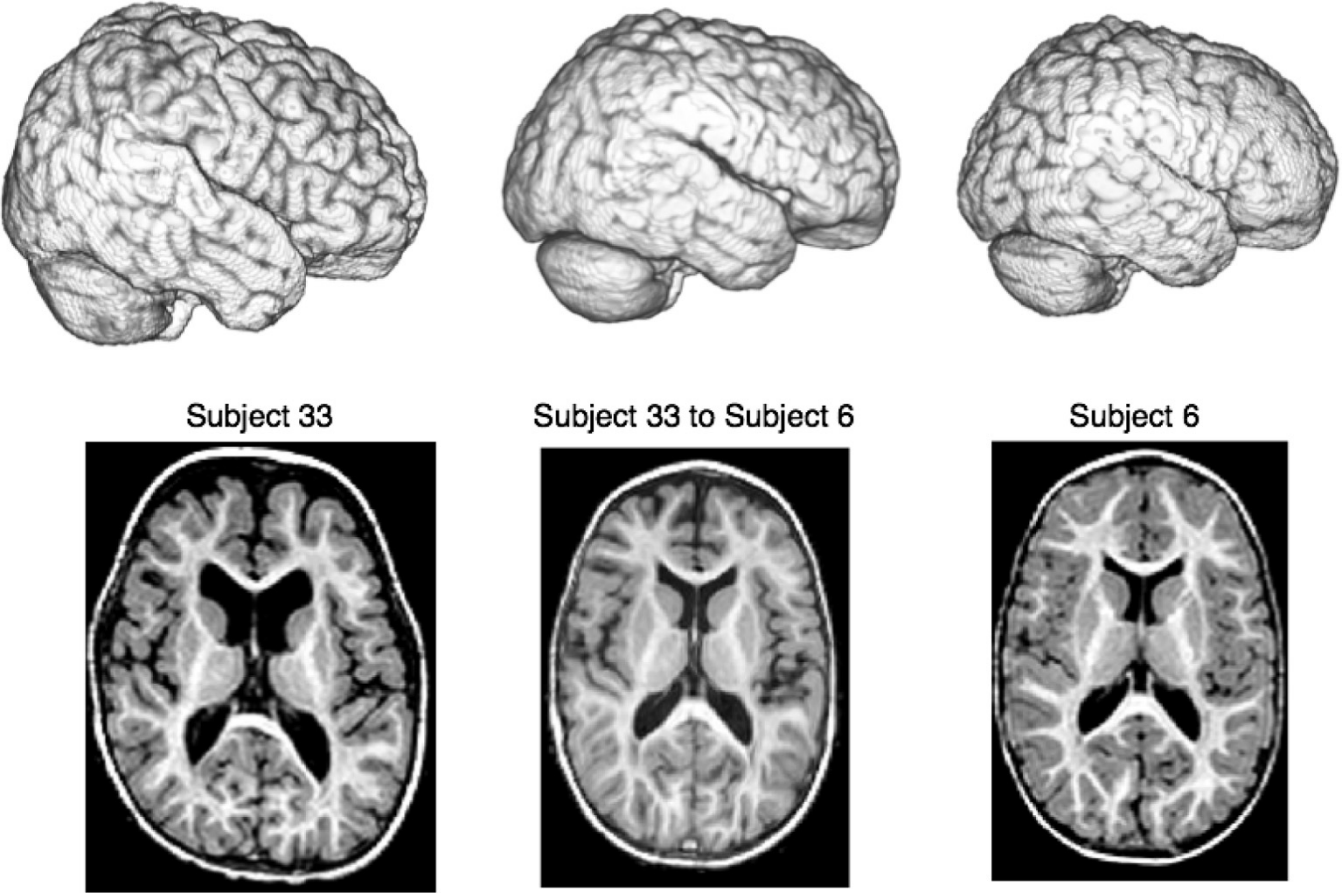
**We compare a ITK^4^ composite schema as *I* ≈_cc_ ↭ ≈_mi_ → *J*_*i*_ for mapping a set of {*J*_*i*_} images to a template *I* to a ITK^3^ schema: *I* ≈_mi_ ⇝_*b*_ ≈_mi_ → *J*_*i*_**. We use this schematic in a registration-based segmentation of multiple brain structures in a pediatric population as a benchmark for algorithm performance, similar to Klein et al. (2010). An example ANTs-based large-deformation result from the dataset is shown for illustration where we render the extracted brains as well as show select axial slices. All registrations were run on the original MRI data with no preprocessing except what is done by ANTs or BRAINSFit internally. Overlap improvement from v3 to v4, quantified via paired *t*-test, is highly significant.

**Figure 7:**
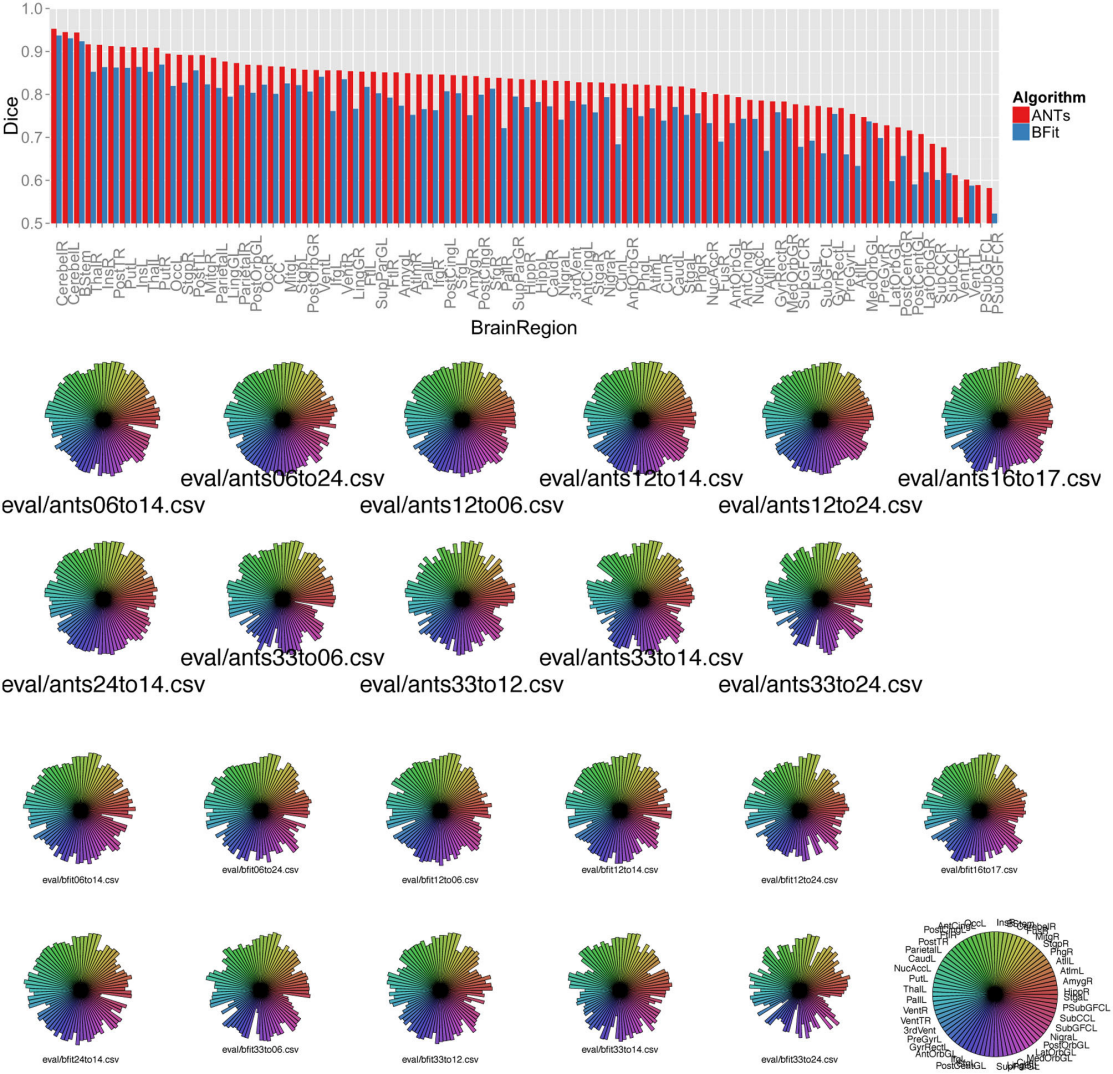
**Above, a barplot shows the mean Dice score for each region and each algorithm, sorted by ANTs performance**. Below, we use star plots of per-brain-region Dice overlap to compare, for each subject, the ITK^4^ implementation of SyN with the ITK^3^-based BRAINSFit algorithm. The ITK^4^ SyN algorithm, with its classic neighborhood correlation metric, outperforms BRAINSFit in several regions and more strongly in some subject pairs than others. The legend for the plots is at lower right and shows the maximum possible value for each region.

As ground truth, we use T1 MRI data from 33 2-year old subjects as described in Gousias et al. (2008) and available at http://www.brain-development.org. Each subject's brain is manually parcellated into 83 distinct regions that include ventricles, cortical areas, white matter and deep gray matter regions such as the amygdala, hippocampus and thalamus. One benefit of this data is that some of these anatomical regions are relatively easy to align (the caudate) whereas others are relatively difficult to align due to their small size (amygdala) or inconsistent shape across subjects (the inferior frontal gyrus). Thus, we anticipate that performance gains due to new technology in ITK^4^ will be most prominent in the more variable and challenging regions. Figure 6 summarizes the study nomenclature and shows a single image pair selected from this data along with the registration result given by ITK^4^. Figure 7 summarizes these evaluation results. The scripts for running this study are available at https://github.com/stnava/ITKv4Documentation/tree/frontiers/scripts. The git hashtag for the ANTs version used in this evaluation https://github.com/stnava/ANTs is ce8b5a7414ae9e389071d756c5f36ee6cecbcfd8. The associated ITK tag is contained within the ANTs repository. The git hashtag for the BRAINSFit version https://github.com/BRAINSia/BRAINSTools used in this evaluation is ad7e114ab1c92bd800819b80e0548259398931c8. Both programs were run on a MacBook Pro running OS X 10.9 (13A3028) with a 2.6 GHz Intel Core i7 and 16 GB of RAM.

To help isolate which subject pairs to deformably register, we first clustered the initial dataset based on an all pairs affine registration which revealed five representative subject clusters. The subject pairings used during evaluation were chosen such that each subject pair contained the most representative subject for one cluster paired with the most representative subject from another cluster; thus, the study design allows us to focus, in a principled manner, on a set of representative shape comparisons where the comparisons are made across different image types.

The new methods in ITK^4^ show enhanced performance within all registration pairs. The mean overall performance gain was approximately 6.3% with a standard deviation of 5% and *T*-statistic/*p*-value, over all structures, of 12.6 / *p* < 1.*e* − 16. We also identified which regions were most improved in ITK^4^ vs. ITK^3^. These regions include the left and right insula, the brainstem, the superior temporal gyrus, parahippocampal gyrus, putamen, and the substantia nigra. Table 1 lists all structures and the mean Dice score for each algorithm, along with the *p*-value. Figure 7 summarizes all of these findings by using star plots to visualize the Dice results for every region in every subject.

**Table 1:** **Dice overlap for ANTs and BRAINSFit where only regions with *q*-value <0.01 are shown**.

	**meanants**	**meanbfit**	**FDR-adjusted**
			***p*-value**
R Hippocampus	0.835	0.770	0.0083
L Hippocampus	0.834	0.782	0.0063
R Amygdala	0.843	0.752	0.0027
L Amygdala	0.851	0.773	0.0037
L AnteriorTemporalLobeMedialPart	0.849	0.752	0.0005
R AnteriorTemporalLobeLateralPart	0.754	0.633	0.0002
L AnteriorTemporalLobeLateralPart	0.786	0.668	0.0083
R Gyri parahippocampalis et ambiens	0.813	0.756	0.0037
L Gyri parahippocampalis et ambiens	0.823	0.749	0.0007
R Superior temporal gyrus posterior	0.892	0.827	0.0001
R Medial and inferior temporal gyri	0.891	0.823	0.0007
R Lateral occipitotemporal gyrus (gyrus fusiformis)	0.801	0.690	0.0023
R Cerebellum	0.953	0.937	0.0066
Brainstem	0.944	0.923	0.0001
R Insula	0.910	0.864	0.0001
L Insula	0.915	0.863	0.0001
L Occipital lobe	0.895	0.819	0.0049
L Posterior temporal lobe	0.912	0.862	0.0001
L Parietal lobe	0.885	0.815	0.0052
R Putamen	0.909	0.869	0.0002
L Putamen	0.911	0.862	0.0003
R Pallidum	0.838	0.721	0.0037
L Pallidum	0.846	0.766	0.0049
L Lingual gyrus	0.876	0.795	0.0037
L Cuneus	0.825	0.684	0.0083
L Lateral orbital gyrus	0.728	0.598	0.0037
L Substantial nigra	0.831	0.741	0.0023
L Superior temporal gyrus anterior part	0.818	0.752	0.0043

We also recorded the amount of processing time spent on each subject, for each algorithm. Noting that the ITK^4^ algorithm also provides a dense and high-resolution forward and inverse transform and does explicit transformation regularization to guarantee a diffeomorphism, the algorithm takes, on average, 5 times as long as the ITK^3^ BRAINSFit algorithm (≈10 min), assuming default settings. Much of this time, in ANTs, is taken up by full resolution image registration. If this fine level is avoided, then the disparity in timing reduces to less than a factor of two, without much loss in accuracy. Note also that ANTs and BRAINSFit each use a different multithreading strategies, similarity metric implementations, rigid/affine registration mechanisms and optimizers making this overall comparison less than ideal.

## 4. Discussion

ITK is a community built and maintained toolkit and is a public resource for reproducible methods. The updated ITK^4^ registration framework provides a novel set of user-friendly parameter setting tools and benchmark implementations of both standard and advanced algorithms. Robustness with respect to parameter settings has long been a goal of image registration and ITK^4^ takes valuable steps toward the direction of automated parameter selection. The primary decision left up to the user, currently, is the feature scale at which registration should be performed. E.g., whether the registration should focus on coarse features, fine features, etc and the different resolutions at which this should be done. While we have provided a reproducible reference comparison of registration-based brain labeling in this paper, we intend to have a more extensive series of benchmark performance studies completed on datasets beyond the brain. However, the number of possible applications exceeds what can possibly be evaluated by the core ITK developer community. Community involvement is needed in order to increase the number of possible registration applications and metric/transform/optimizer/data combinations that have been evaluated. At the same time, documentation, usability and examples must be provided by the development team in order to improve user involvement.

### 4.1. Future work

Future work will enhance the depth and breadth of documentation as well as seek to further optimize the current implementations for speed and memory. In time, it may be possible to extend the design philosophy used here to GPU implementations. However, our ability to interface low and high-dimensional transformations depends heavily on generic programming. This style is less well-developed (and less well understood) in GPU applications which depend, to some extent, on specialization. The current framework is amenable to groupwise registration strategies when used in combination with a computing cluster. However, single core groupwise strategies are not currently implemented although one may consider basing an implementation on exisiting multi-metric/multivariate registration tools within the current code base. While ITK^4^ does contain a statistics infrastructure, we currently prefer using *R* and *ANTsR* for analyzing our data. However, the lack of visualization methods in ITK means that one must still move to another package to look at one's results. Therefore, direct interfaces to *R* remain useful. SimpleITK also has a promising *R* interface that is similar to *ANTsR*.

A primary challenge to the future of ITK^4^ includes, beyond documentation, reduced *C*++ fluency. As ITK^4^ leverages several advanced features of *C*++, even experienced developers may find it difficult to contribute meaningfully to the ITK software base. Therefore, the ITK^4^ community must also seek to educate potential future contributors not only on ITK but also, at times, on the fundamentals or advanced extensions of *C*++. A second major hurdle is that ITK^4^ includes a host of generic registration ingredients. However, many of the most compelling new application domains require specialization. Specialization may be needed for a specific imaging modality, via hardware interface or in the use of domain-specific prior knowledge. Therefore, we envision the next phase of ITK^4^ development may focus on using the toolkit to support its specialization in solving high-impact and translational applications. Hopefully, this transition will occur in the near future.

### Conflict of interest statement

The authors declare that the research was conducted in the absence of any commercial or financial relationships that could be construed as a potential conflict of interest.

## Footnotes

^2^All ITK^4^ metrics are set to be minimized. For instance, the itkMattesMutualInformationImageToImageMetricv4 returns negative mutual information.

^3^In the Demons example above, the reader may ask: why does the affine mapping, *A*, not appear “inside” the deformable mapping, ϕ? Indeed, this ordering of transformations is feasible. However, this is not what we typically use in our own practice of image registration and is not what we recommend. The reason is that we usually seek a deformable mapping into a template space that is common across many subjects (i.e., the “tail” is in the same domain across subjects). This enables methods such as Jacobian-based morphometry and other groupwise comparison conveniences. For example, see Figures 1 through 4 in (Kim et al., 2008) which explains the classical approach of Jacobian-based morphometry as applied to traumatic brain injury. See also (Gaser et al., 2001; Riddle et al., 2004; Dubb et al., 2005; Lepore et al., 2006; Rohlfing et al., 2006; Studholme and Cardenas, 2007). Additional uses of Jacobian-based morphometry are shown in our examples section in the “C” example and the “asymmetry” example.
